# Supplementary material for: Multiplicative priming of the correct response can explain the interaction between Simon and flanker congruency
Source: PLoS One. 2021 Mar 9;16(3):e0248172. doi: 10.1371/journal.pone.0248172 (PMC7943002; doi:10.1371/journal.pone.0248172)
Supplement: S1 File — (PDF) [file pone.0248172.s001.pdf]

S1 File

Performance on the pure Simon and flanker blocks

Table S1a. Experiment 1: Inferential Statistical Values for the Follow-Up *t*-Tests Comparing Incongruent and Congruent Trials (Congruency Effect), Incongruent and Neutral Trials (Interference Effect) as well as Neutral and Congruent Trials (Facilitation Effect), and Bayes Factors (BF) from Model Comparisons.

Exp-eriment	Block type	Difference	Reaction times					Arcus-sinus transformed error rates				
			t-test			Bayesian *t*-test		*t*-test			Bayesian *t*-test	
			t	*p*	Cohen's *d*	BF_{10}	BF_{01}	*t*	*p*	Cohen's *d*	BF_{10}	BF_{01}
1a	Simon	Incongruent vs. congruent	8.71	<.001	1.62	6.28×10^{6}	1.59×10^{-7}	5.73	<.001	1.06	5.15×10^{3}	1.94×10^{-4}
		Incongruent vs. neutral	12.25	<.001	2.28	8.65×10^{9}	1.16×10^{-10}	2.70	.012	0.50	4.01	0.25
		Neutral vs. congruent	0.73	.471	0.14	0.25	3.97	4.33	<.001	0.80	160.16	6.24×10^{-3}
	Flanker	Incongruent vs. congruent	5.71	<.001	1.06	4.94×10^{3}	2.03×10^{-4}	1.62	.115	0.30	0.64	1.57
		Incongruent vs. neutral	4.56	<.001	0.85	283.63	3.53×10^{-3}	2.04	.051	0.38	1.19	0.84
		Neutral vs. congruent	1.90	.068	0.35	0.95	1.05	-0.85	.404	0.16	0.27	3.65
1b	Simon	Incongruent vs. congruent	9.95	<.001	1.82	1.32×10^{8}	7.57×10^{-9}	7.27	<.001	1.33	2.73×10^{5}	3.67×10^{-6}
		Incongruent vs. neutral	-0.73	.472	0.13	0.25	4.03	3.69	<.001	0.67	35.41	0.03

Exp-eriment	Block type	Difference	Reaction times					Arcus-sinus transformed error rates				
			t-test			Bayesian *t*-test		*t*-test			Bayesian *t*-test	
			t	*p*	Cohen's *d*	BF_{10}	BF_{01}	*t*	*p*	Cohen's *d*	BF_{10}	BF_{01}
		Neutral vs. congruent	10.26	<.001	1.87	2.60×10^{8}	3.84×10^{-9}	4.60	<.001	0.84	331.57	3.02×10^{-3}
	Flanker	Incongruent vs. congruent	8.06	<.001	1.47	1.86×10^{6}	5.39×10^{-7}	-0.96	.346	0.17	0.30	3.38
		Incongruent vs. neutral	1.33	.193	0.24	0.43	2.31	-0.63	.531	0.12	0.23	4.27
		Neutral vs. congruent	5.62	<.001	1.03	4.39×10^{3}	2.28×10^{-4}	0.04	.972	0.01	0.19	5.14

*df*s = (28) and *df*s = (29) for the *t* statistic in Experiment 1a and 1b, respectively. BF_{10} = Bayes Factor in favor of the alternative hypothesis (i.e., in favor of the effect). BF_{01} = Bayes Factor in favor of the null hypothesis (i.e., in favor of the absence of the effect).

Table S1b. Experiment 1: Inferential Statistical Values for the One-Way Repeated-Measures Analysis of Variance (ANOVA) with the Variable Congruency (Incongruent, Neutral, Congruent), and Bayes Factors (BF) from Model Comparisons.

Exp-eriment	Block type	Reaction times						Arcsine transformed error rates					
		ANOVA				Bayesian analysis		ANOVA				Bayesian analysis	
		df	*F*	*p*	η_g^2	BF_{10}	BF_{01}	*df*	*F*	*p*	η_g^2	BF_{10}	BF_{01}
1a	Simon	1.67, 46.66	63.11	<.001	.07	7.49×10^{11}	1.34×10^{-12}	1.82, 50.90	21.92	<.001	.11	1.20×10^{5}	8.32×10^{-6}
	Flanker	1.94, 54.38	19.52	<.001	.02	3.31×10^{4}	3.02×10^{-5}	1.85, 51.84	2.76	.077	.01	0.84	1.20
1b	Simon	1.99, 57.80	67.25	<.001	.12	4.09×10^{12}	2.44×10^{-13}	1.93, 55.93	31.51	<.001	.18	1.47×10^{7}	6.81×10^{-8}
	Flanker	1.94, 56.19	30.46	<.001	.06	8.32×10^{6}	1.20×10^{-7}	1.58, 45.70	0.38	.635	.003	0.13	7.42

Effect sizes are expressed as generalized η^2 values. BF_{10} = Bayes Factor in favor of the alternative hypothesis (i.e., in favor of the effect). BF_{01} = Bayes Factor in favor of the null hypothesis (i.e., in favor of the absence of the effect).

Table S1c. Experiment 2: Inferential Statistical Values for the Two-Way Analysis of Variance (ANOVA) with Congruency (Incongruent, Congruent) as a Within-Subject Variable and Position (Simon Horizontal – Flanker Vertical, Simon Vertical – Flanker Horizontal) as a Between-Subjects Variable, and Bayes Factors (BF) from Model Comparisons.

Block type	Effect – Model	Reaction times					Arcsine transformed error rates				
		ANOVA			Bayesian analysis		ANOVA			Bayesian analysis	
		F	*p*	η_g^2	BF_{10}	BF_{01}	*F*	*p*	η_g^2	BF_{10}	BF_{01}
Simon	Congruency	136.09	<.001	.08	9.88×10^{11}	1.01×10^{-12}	47.55	<.001	.12	5.35×10^{5}	1.87×10^{-6}
	Position	0.57	.454	.01	0.56	1.78	0.37	.546	.01	0.39	2.54
	Congruency x Position	2.07	.157	.001	0.64	1.57	1.88	.177	.01	0.61	1.64
Flanker	Congruency	173.78	<.001	.04	1.59×10^{13}	6.28×10^{-14}	14.89	<.001	.02	76.65	0.01
	Position	0.30	.587	.006	0.61	1.64	0.32	.573	.01	0.54	1.84
	Congruency x Position	6.06	.018	.001	2.85	0.35	0.01	.944	<.001	0.28	3.52

*df*s = (1, 46) for the *F* statistic. Effect sizes are expressed as generalized η^2 values. BF_{10} = Bayes Factor in favor of the alternative hypothesis (i.e., in favor of the effect). BF_{01} = Bayes Factor in favor of the null hypothesis (i.e., in favor of the absence of the effect). As the interaction between Congruency and Position was significant in the reaction times for the pure flanker blocks, we carried out follow-up *t*-tests. These showed a smaller – but still significant – flanker congruency effect in the “Simon vertical – flanker horizontal” condition (39 ms), t (23) = 8.90, $p < .001$, $d = 1.82$, $BF_{10} = 1.90 \times 10^{6}$, $BF_{01} = 5.25 \times 10^{-7}$, than in the “Simon horizontal –

flanker vertical” condition (56 ms), t (23) = 9.80, $p < .001$, $d = 2.00$, $BF_{10} = 9.96 \times 10^{6}$, $BF_{01} = 1.00 \times 10^{-7}$.

Table S1d. Experiment 3: Inferential Statistical Values for the *t*-Test Comparing Incongruent and Congruent trials, and Bayes Factors (BF) from Model Comparisons.

Block type	Reaction times					Arcsine transformed error rates				
	t-test			Bayesian *t*-test		*t*-test			Bayesian *t*-test	
	t	*p*	Cohen's *d*	BF_{10}	BF_{01}	*t*	*p*	Cohen's *d*	BF_{10}	BF_{01}
Simon	9.37	<.001	1.56	2.16 x 10^{8}	4.63 x 10^{-9}	6.01	<.001	1.00	2.28 x 10^{4}	4.38 x 10^{-5}
Flanker	8.14	<.001	1.36	8.50e x 10^{6}	1.18 x 10^{-7}	0.34	.733	0.06	0.19	5.29

*df*s = (35) for the *t* statistic. BF_{10} = Bayes Factor in favor of the alternative hypothesis (i.e., in favor of the effect). BF_{01} = Bayes Factor in favor of the null hypothesis (i.e., in favor of the absence of the effect).

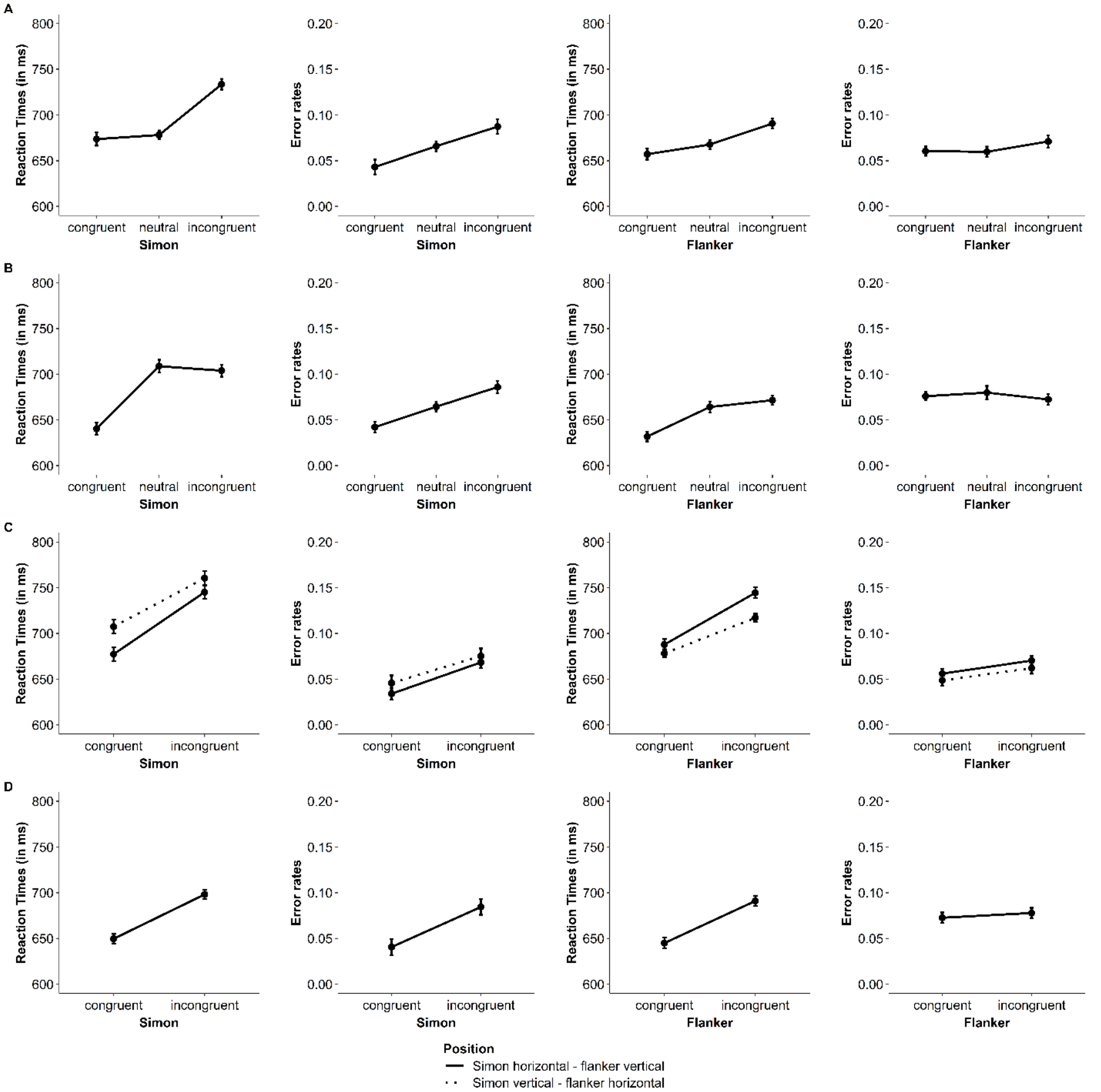

Fig S1. Performance on the pure blocks: Mean reaction times and mean raw error rates for both Simon congruency (left part) and flanker congruency (right part). Error bars represent within-subject confidence intervals [see 39,40]. (A) Experiment 1a. (B) Experiment 1b. (C) Experiment 2. (D) Experiment 3.
