# Supplementary material for: Multiplicative priming of the correct response can explain the interaction between Simon and flanker congruency
Source: PLoS One. 2021 Mar 9;16(3):e0248172. doi: 10.1371/journal.pone.0248172 (PMC7943002; doi:10.1371/journal.pone.0248172)
Supplement: S2 File — (PDF) [file pone.0248172.s002.pdf]

S2 File

Delta plots

Delta plots show the change in the magnitude of the congruency effect as a function of response speed. The y-axis of a delta plot shows the size of the congruency effect at each percentile, whereas its x-axis shows the RT performance averaged across incongruent and congruent trials (see [51] for an example). To illustrate the interaction between congruency variables, two types of delta plots are presented for each experiment. First, a delta plot was computed for the flanker congruency effect depending on whether the trials were Simon incongruent or congruent (left part in each figure of S2 File). Second, a delta plot was computed for the Simon congruency effect depending on whether the trials were flanker incongruent or congruent (right part in each figure of S2 File). Delta plots for Experiment 1 are presented in Fig S2a (incongruent trials vs. congruent trials), in Fig S2b (incongruent trials vs. neutral trials) and in Fig S2c (congruent trials vs. neutral trials). Delta plots for Experiment 2 are presented in Fig S2d. Delta plots for Experiment 3 are presented in Fig S2e.

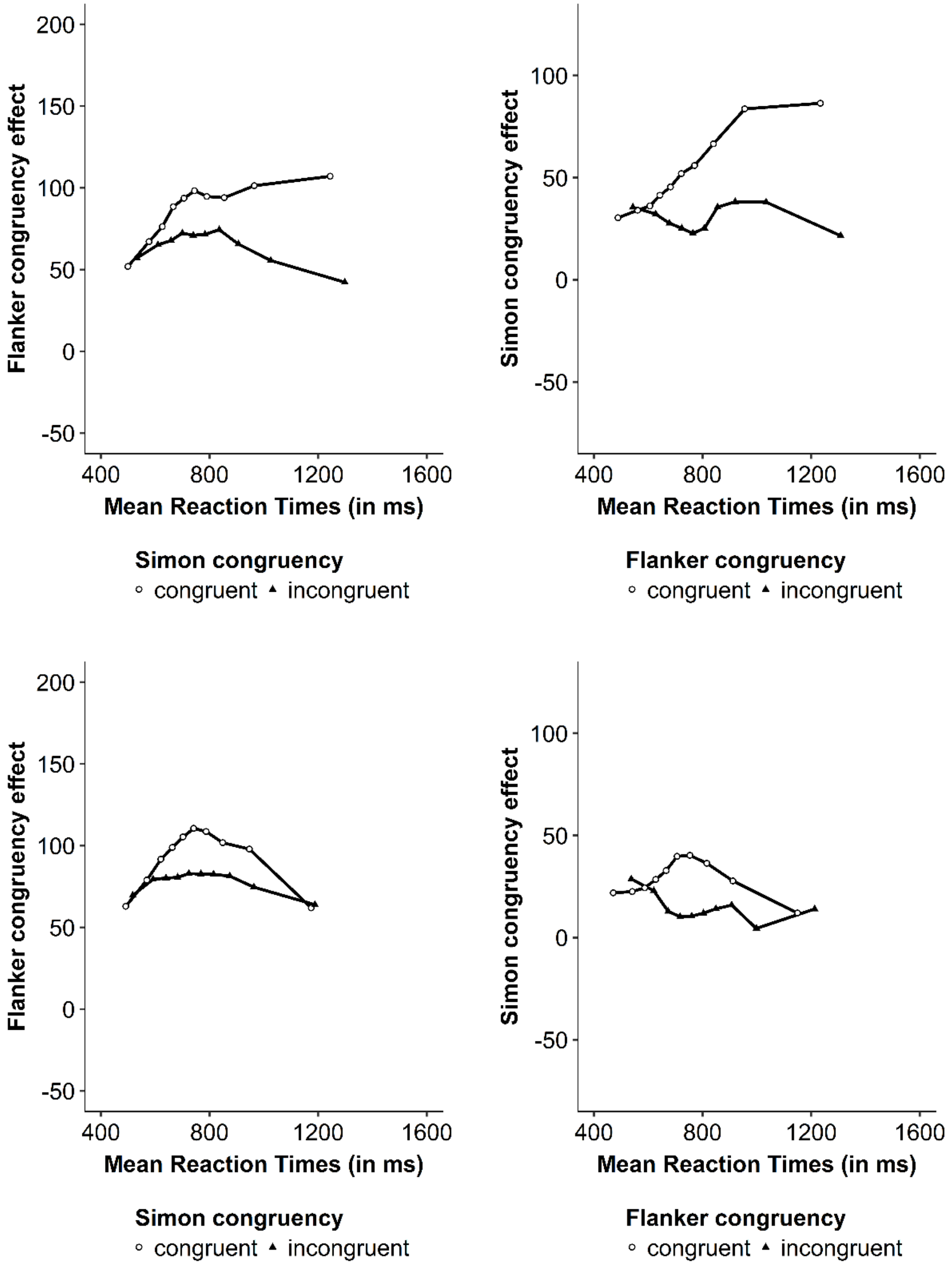

Fig S2a. Experiment 1: Congruency effect (incongruent vs. congruent). Delta plots for the flanker congruency effect (left part) and the Simon congruency effect (right part). Reaction times are given in milliseconds (ms). Top part: Experiment 1a. Bottom part: Experiment 1b.

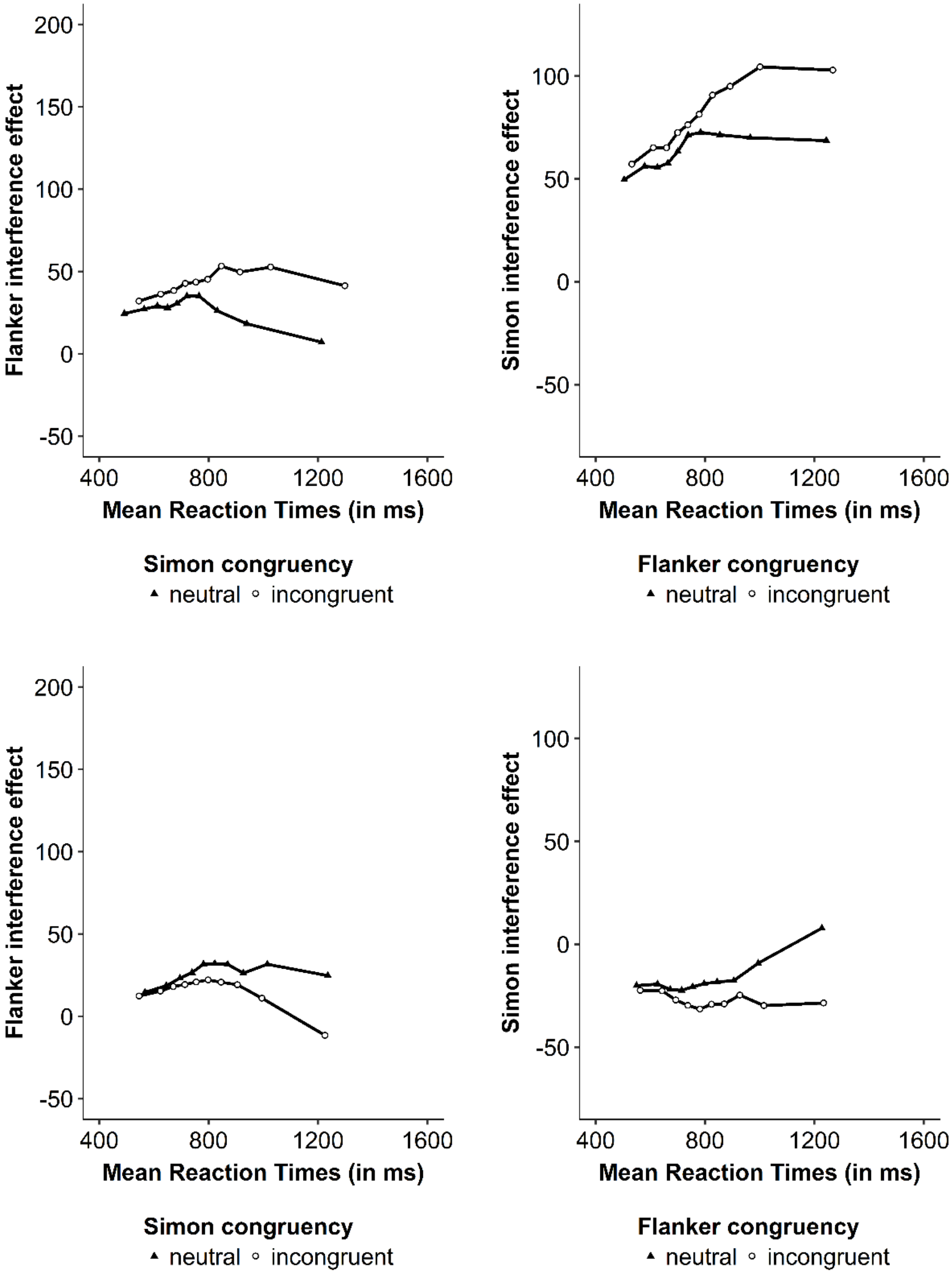

Fig S2b. Experiment 1: Interference effect (incongruent vs. neutral). Delta plots for the flanker interference effect (left part) and the Simon interference effect (right part).

Reaction times are given in milliseconds (ms). Top part: Experiment 1a. Bottom part: Experiment 1b.

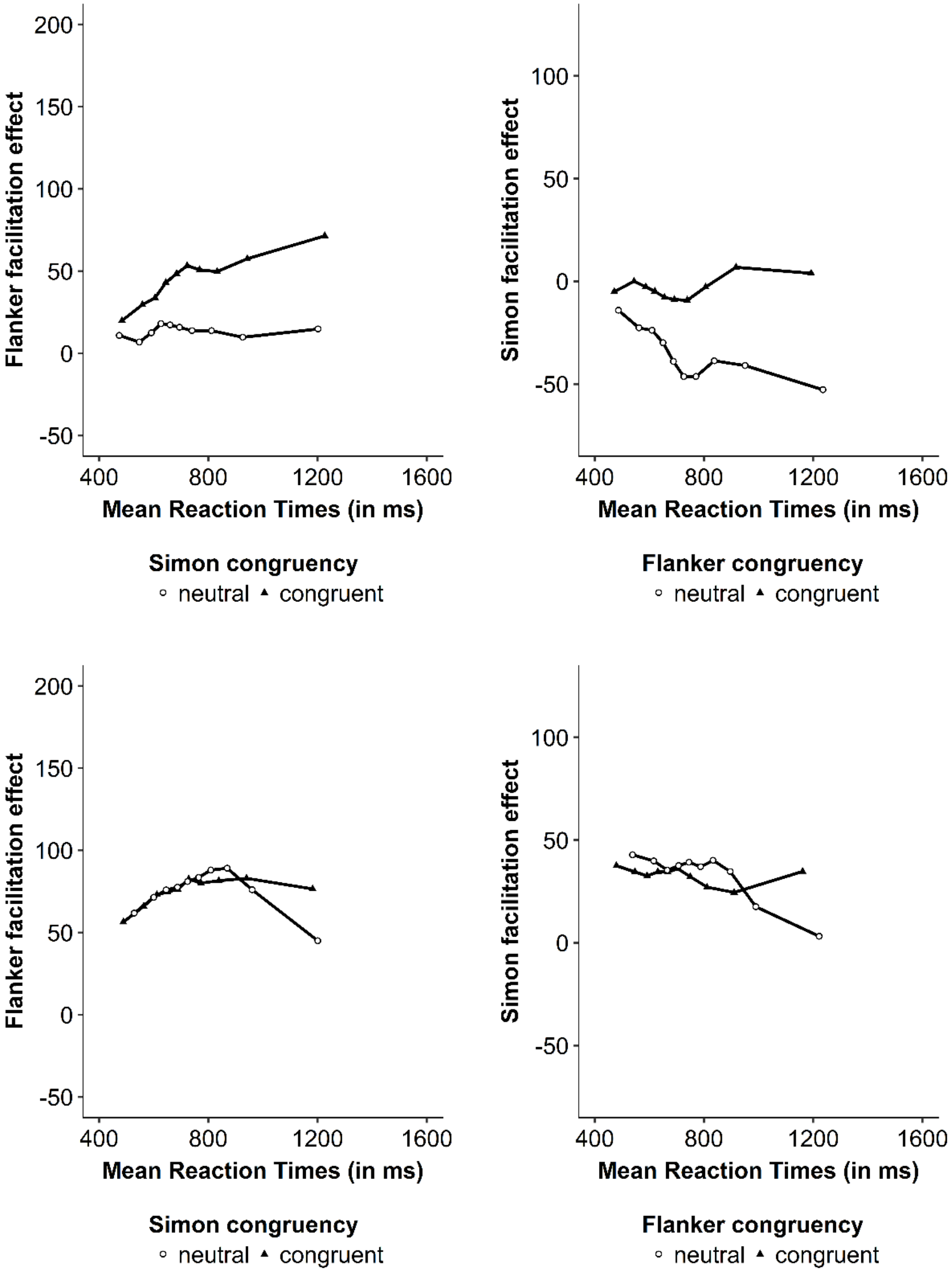

Fig S2c. Experiment 1: Facilitation effect (neutral vs. congruent). Delta plots for the flanker facilitation effect (left part) and the Simon facilitation effect (right part). Reaction times are given in milliseconds (ms). Top part: Experiment 1a. Bottom part: Experiment 1b.

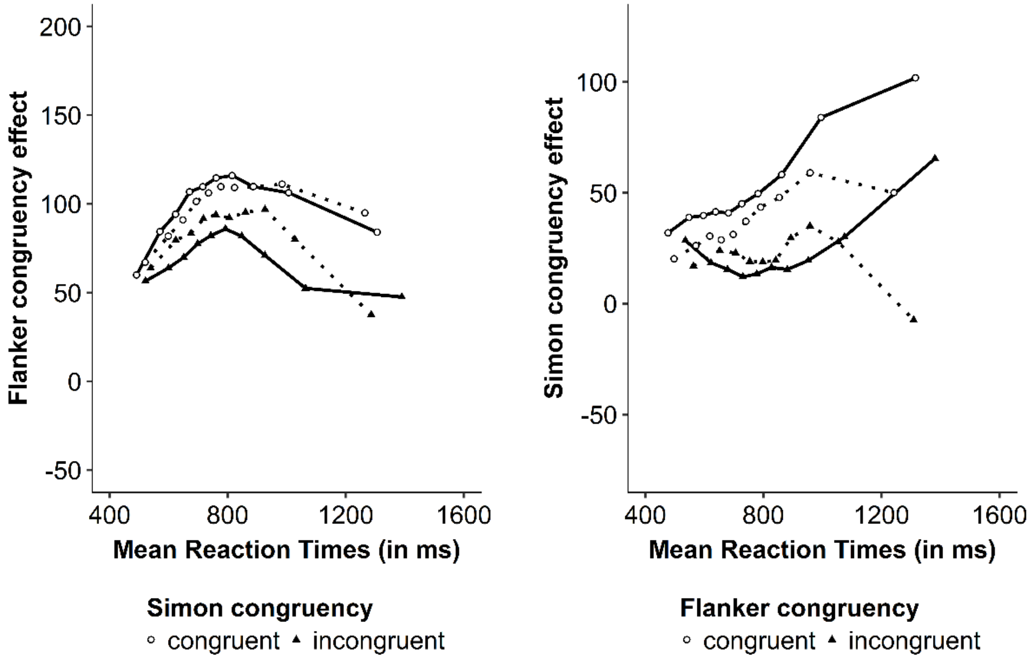

Fig S2d. Experiment 2: Delta plots for the flanker congruency effect (left part) and the Simon congruency effect (right part). Solid lines represent the Position "Simon Horizontal – Flanker Vertical", and dotted lines represent the position "Simon Vertical – Flanker Horizontal". Reaction times are given in milliseconds (ms).

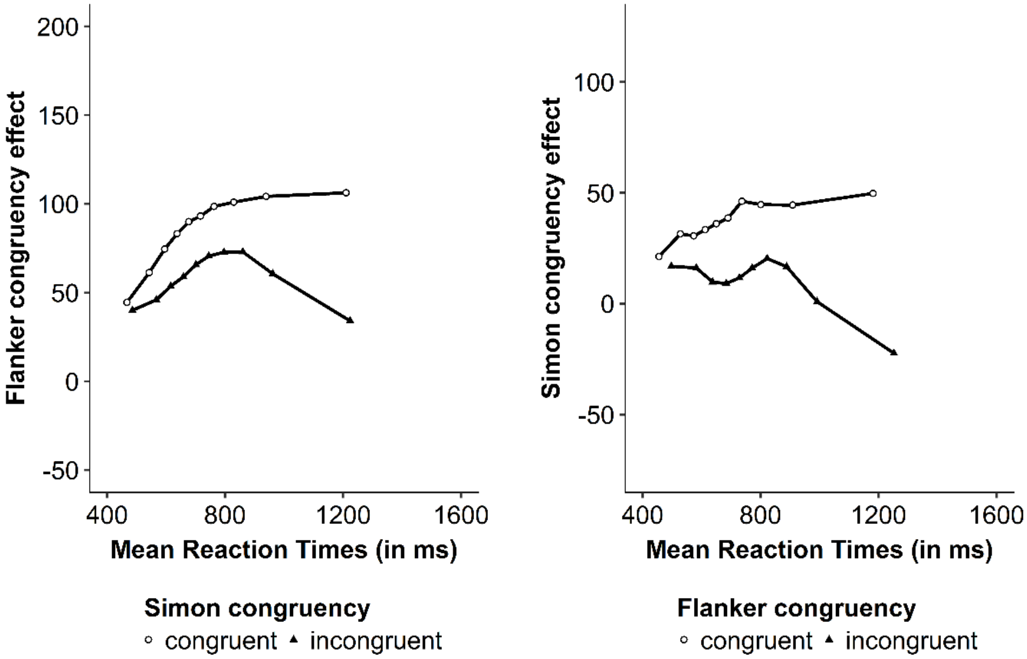

Fig S2e. Experiment 3: Delta plots for the flanker congruency effect (left part) and the Simon congruency effect (right part). Reaction times are given in milliseconds (ms).
