# Supplementary material for: Multiplicative priming of the correct response can explain the interaction between Simon and flanker congruency
Source: PLoS One. 2021 Mar 9;16(3):e0248172. doi: 10.1371/journal.pone.0248172 (PMC7943002; doi:10.1371/journal.pone.0248172)
Supplement: Attachment — Submitted filename: Comments (ROUND II) upload.pdf [file pone.0248172.s005.pdf]

The authors have taken into consideration many of the points I raised in the previous round of review. I can now better understand (and evaluate) the literature they mention, their line of reasoning and their claims. In my view, the narrative of the manuscript has improved, but there are still many inconsistencies and questionable points, which, however, I think the authors could fix.

1) In my view, one of the most questionable points concerns the "tentative" account the authors propose at the end of the General Discussion to reconcile their results with some of the previous findings. To do so, in fact, they propose an account that is inconsistent with many other findings and with the main hypotheses about how responses are selected in this type of compatibility tasks.

Forgive me for the long explanation below, but I need to be sure that the authors and I refer to the same theoretical framework.

The interaction between two factors that likely affect the same (response selection) stage (as it is the case with Simon and flaker compatibilities) is thought to depend on (a) the overlap in time between the effect of the processing of one irrelevant attribute and the effect of the processing of the other irrelevant attribute, and (b) on the overlap of the processing of these attributes with the processing of the relevant attribute and the controlled-route activation of the correct response (see, e.g., Gevers, Caessens, Fias, EJCP, 2010; Kornblum, Stevens, Whipple, & Requin, 1999; Ridderinkhof et al.,2004). The overlap or the lack of overlap between these processes are not fixed properties of a given paradigm (e.g., Simon paradigm), but they depend on the particular structure of the specific task that is used and on the relevant and irrelevant stimulus features that the task involves: some features (e.g., colors) can be processed and discriminated before others (e.g. letters, direction of arrows).

Usually, in standard horizontal Simon tasks, the Simon effect is observed at the first bins of RT distributions and it is not observed when the relevant feature is relatively difficult to discriminate (and, thus, when responses are relatively slow). This has been explained on the basis of a temporal overlap account (Hommel 1994). The spatial code in standard Simon tasks is thought to be formed quite early: immediately after stimulus onset (e.g., indeed, according to the attention-shift account of the Simon effect, stimulus spatial coding occurs because attention has to move towards he stimulus and thus the irrelevant spatial code is formed even before the *relevant* stimulus feature is analysed). Afterward, the spatial code decays. Therefore, this code can have an effect (i.e., it can speed up RTs wen the primed response corresponds to the correct one or lengthen RTs wen the primed response is wrong, and thus needs to be inhibited) only if the activation of the correct response by the relevant attribute occurs shortly after, that is, only in the case of relatively fast responses. Response priming mediated by the automatic routes, the correct-response activation mediated by the controlled route, and the conflict between them, have been shown to occur **at reponse/motor related stages** that can be tapped through techniques assessing either muscle or motor cortex activation: lateralized readiness potentials (De Jong et al., 1994) and EMG recordings show an initial activation of the spatially corresponding response (shortly after stimulus presentation) followed by a decrease of such activation when this response is the incorrect one and, finally, by the activation of the correct response (see, e.g., Treccani, Cona, Milanee, Umiltà, Psych. Res, 2018 for a review).

The irrelevant flanker feature (the one that the flanker has in common with the target) is thought to exert its effect on the same processing stage as the irrelevant spatial code (i.e., the response selection stage) but it usually exert its effects later – indeed, the coding of the irrelevant flanker attribute cannot precede the processing of the target relevant feature, as happens instead in the case of stimulus location. Response-priming triggered by the flanker can overlap in time with the (controlled) activation of the correct response but, typically, we observe an effect of the former on the latter (i.e., we observe, a flanker effect) only in later bins compared to those in which we observe the Simon effect, and typically we observe that the flanker congruency effect increases as RTs increase. Therefore, when the two tasks are combined in a flanker-Simon tasks, one should expect that, most of the times, the effects of the two factors do not overlap in time and they do not interact with each other (see, e.g., Gevers et al., 2010, for a similar explanation for either the interaction or lack of interaction between Simon and SNARC effects). This may explain why in many studies no significant interaction between Simon and flanker effects was found.

However, this is not a fixed rule. If, for example, the task is such as to allow a very fast processing of the feature shared by the target and flanker, or to *prevent* a fast processing of the irrelevant spatial feature leading to the Simon effect, than an overlap between the effects of these processes (and a significant interaction) can be observed (cf., e.g., Gevers et al., 2010 for the modulation of the interaction between

Simon and SNARC that either emerged or did not emerge according to the feature that participants had to judge)

The accessory-stimulus Simon task used by Treccani et al. is an example of a task in which the Simon effect increases as RTs increase, just as the flanker effect (and this is a quite sensible explanation of why in their case the overlap occurs and a significant interaction was found – see also my 'other points' below) but similar temporal patterns have been obtained with other tasks that are not the standard one.

For example, stable or increasing Simon effect functions have been also obtained for vertical Simon tasks, that is, tasks in which stimuli and responses are aligned vertically rather than horizontally (Vallesi et al, Cognition, 2005; Wiegand & Wascher, Ps. Res., 2007). By using these tasks combined with a flanker task, one should thus expect an interaction between the Simon and flanker effects. Interestingly, in some of the experimental tasks used here, stimuli and responses were aligned vertically (i.e., the task was a sort of vertical Simon task). In fact, even the "horizontal" tasks administered here were clearly not standard Simon tasks and we could expect the temporal trend of the Simon effect to be different from the typical one.

Indeed, in their experiments, the authors DO NOT observe the typical decreasing Simon effect functions: they found that "*the Simon congruency effect initially increased while the RTs increased from fast to moderate*".

This means that, in the present study, there was indeed a temporal overlap between the Simon and flanker effects (i.e., the effect of the stimulus location and of the flanker non-spatial attribute), which is the prerequisite for an interaction between the two effects to occur.

Accordingly, I think that there is no need to add ad-hoc assumptions to explain why an interaction was found here.

For example, the authors propose that: "*A tentative way to explain these findings is that that the interaction does not emerge during conflict resolution, but appears on the level of motor responses*". However, the way, in which the authors explains the multiple- priming hypothesis clearly refers to a stage that previous research has identified as the stage in which motor priming AND response conflict can occur: there cannot be a stage in which response conflict occurs and a stage in which responses are primed, at least following the current models of response selection. The conflict in incongruent trials occurs because some responses are primed and these responses are not the correct ones. The authors also maintain that "*…the flankers and the irrelevant location could activate their respective motor responses via direct associations already during early stimulus processing*"-> This is indeed plausible and it is consistent with many models of response section: the response/motor related stage (in which responses are automatically primed by irrelevant information, while others are chosen, via controlled routes, on the basis of the relevant features) does not need to start after stimulus processing is completed, but this does not mean that there is such a thing as a stage in which response priming occusa and another stage in which response is selected. "*Thus, the flanker condition is first processed, pre-activating the correct response. Then, the Simon condition is processed, pre-activating the correct response again. These pre-activations of motor responses then result in a co-activation of the correct response at the stage of response selection*" > The flanker or Simon "conditions" cannot (as a rule) activate the correct response. Maybe, the authors meant that the flanker irrelevant feature pre-activates the associated response, which, in flanker congruent trials, is the correct response and the target position pre-activates the associated response which, in Simon congruent trials, is the correct response. Again, this activation (response/motor priming) is part of the response selection processes and is what is thought to cause conflict or facilitation at the response selection stage.

2) I still find the "bias in learning" account not very convincing. For example, the authors claim "*In particular, the stimulus-response pairings from the congruent-congruent trials are presented more often than those of any other trial types. Thus, because this trial type is presented most frequently, there is more chance that participants learn to assign a response to each stimulus exemplar*", which would result in this type of trials leading to the best performance.

Following the same line of reasoning, one may claim that the stimulus-response pairings from the incongruent-incongruent trials are presented less often than those of any other trial types, which would result in this type of trials leading to the worst performance and imply additivity between flanker and Simon congruencies.

Similarly, the authors claim that "*there is no a priori reason to prefer a linear learning process*", that is, a trend that would result in additivity, rather than in an interaction. Yet, there is no reason to think that learning follows an exponential growth curve either (i.e., a trend that would result in an under-additive

interaction) or, vice-versa, a logarithmic growth curve, or, even, a curve with an initial steep slope followed by a plateau (i.e., trends that would result in an over-additive interaction). It depends on the shape of the learning curve and on the part of the curve that is tapped by this particular task (the steep or flat part). I do not know whether there are studies on the learning curve with this kind of material (if yes, the authors may consider mentioning such studies).

Anyway, I suggest the authors claim, more simply, that when the values of the irrelevant stimulus dimensions are more than two, there are different numbers of exemplars (S-R pairings) for the different trial types, which, in turn, leads to different frequencies of presentation of these pairings. The resulting learning effect (better performances for more frequent, and thus more practiced, pairings) might shape the Simon – flanker congruency interaction in unpredictable ways. One of the possible ways in which learning may affect performance results in an under-additive interaction between the two congruency factors: this would occur when more practice does not lead, at first, to better performance (e.g., going from the few presentations of each of the incongruent-incongruent S-R pairings to the relatively more frequent incongruent-congruent S-R pairings) but a significant improvement can be found with much more practice (e.g., with the congruent - congruent S-R parings)

P. 10. "*the central assumption put forward in both accounts [within-trial conflict monitoring and spontaneous decay accounts] has been put into question by a study using ERPs [...]the results suggest that although flanker conflict and Simon conflict are resolved at separable stages, there appears to be little influence of the earlier stage on the later one*"-> This is misleading: if it were true (two different stages for the two effects), then the multiplicative priming account too should be rule out. According to this account, that the two irrelevant features of the stimulus display (the position and the feature that the flanker shares with the target) affect the same processing stage (the one in which automatic response priming and controlled response activation occur, responses competes and the correct one is selected). Indeed, there is plenty of literature (about 50 years of studies) showing that the Simon and flanker effect emerge at the same stage (the response selection stage), thus I would not claim that the two effects emerge at different stages (this claim would require a long discussion). Actually, evidence showing that the two effect emerge at the same response selection stage is also the main evidence challenging the temporal-overlap account proposed by Hommel for the interaction between the two effects. According to Hommel (1997), the two effects emerge at different stages: (stimulus-encoding and response-selection stages).

3) P. 17 lines 327-342. This part is very difficult to read and maybe also not very useful. The whole thing can be explained in a more simple way. The authors wanted to evaluate if and how an effect can modulate the other and they want to evaluate both the facilitation and interference components, which is a quite sensible strategy here.

4) P. 31. "*Together, these findings support the explanation that the interaction between Simon and flanker congruency results from the multiplicative priming occurring when the trials were congruent for both Simon and flanker conditions*". In my view, these results suggest that the interaction can be found even with this paradigm and that the modulation of one effect on the other mainly involve the facilitation component. I do not see these results of the first two experiments as evidence of the multiplicative priming account. Actually, I do think that neither these experiments nor the following ones directly test this account. This study simply shows that the Simon-flanker interaction is a robust phenomenon, that is, it is not a phenomenon that can only be observed in some peculiar paradigms (the authors observed the interaction in many different experiments - the evidence is strong) and it is not modulated by many of the factors that one may think of as critical factors. The multiplicative priming account appears to be a plausible account for these results (i.e., it is consistent with the pattern of results of the study). The study also offers some useful ideas about how to test this account in future studies. In my view, these are the only fair and reasonable conclusions that can be drawn from the results (and they are not trivial at all).

5) P. 31 lines 599-607. "*It might be surprising that for the Simon condition we observed a reverse facilitation effect*". In my view, this is not surprising at all: That may happen when, in neutral trials, stimuli are presented at fixation, that is, where visual acuity is higher (cf., e.g., Umiltà, Rubichi & Nicoletti, 1999).

The benefit of stimuli being presented at fixation in neutral trials may be greater than the facilitation produced by the irrelevant stimulus location in spatially congruent trials. .
It is also worth noting that it is not uncommon to find a reverse Simon effect in task combing the Simon paradigm with other types of compatibility paradigms (see.,e.g., the many studies about the logical-recoding mechanisms in tasks combining Simon and symbolic compatibility or Keus & Schwarz, 2005, for a task combining Simon and SNARC paradigms)

OTHER POINTS_________
P.:3 _________
- " *In both Simon and flanker tasks, trials are labelled incongruent when they contain features for two different response alternatives* -> .maybe better "when they contain features pointing to two different response alternatives"
-"*a trial is incongruent in the Simon task when the location on the screen is different from the response location*" To be consistent with the sentence above, one should specify that "a trial is incongruent in the Simon task when the stimulus location on the screen activates a response that is different from the correct response".
- "*In the flanker task, a trial is incongruent when the color of the central letter is different from* "'Again, to be consistent with the way in which the authors describe the incongruent condition in the two tasks, one should say that "In the flanker task, a trial is incongruent when the correct response (i.e., the response activated by the color of the central letter) is different from that activated by the color of the flanking..." I also suggest modifying the description of the congruent conditions accordingly.

P. 4____________
- "*Findings from correlational studies showed, however, small to moderate correlations between both congruency effects*". I would mention, however, that many studies show that there small to moderate correlations even between the congruency effects observed in two different administrations of the very same task (cf., e.g., Draheim, Mashburn, Martin, & Engle, 2019)

"*indicating that the congruency effect of one condition (e.g., the flanker condition) was smaller when the trials of the other condition (in this case, the Simon condition)".* I would say: "indicating that one congruency effect (e.g., the flanker effect) was smaller in trials that were incongruent with respect to the other congruency factor (in this case, Simon congruency)

P. 7__________
"*Previous research has used different theoretical accounts to explain the interaction between* Simon and *flanker congruency. A first explanation is based on the conflict monitoring framework* [19]" -> this gives the wrong impression that the interaction between Simon and flanker congruencies has been already explained on the basis of the conflict   monitoring account and that Botvinick et al. ([19]) were those who proposed this explanation of the interaction. In contrast, as I understood it, the authors themselves are those who proposed that this interaction may be explained by this account. In a previous paper, they proposed a similar account for other types of interaction between congruency factors. This should be explained more clearly. For example: "Previous research has used different theoretical accounts to explain the interaction between congruency factors. As proposed for other types of interaction [7], the underadditive interaction between Simon and flanker congruency could be accounted for by the conflict monitoring hypothesis. Following this hypothesis, the conflict monitoring.... accordingly [19]"

Here and in the other parts of the manuscript I would not refer to Simon, flanker or Stroop congruency as "congruency conditions," bur rather "congruency factors". In general, the term "conditions" is used to refer to levels of a factor (i.e., an independent variable.) For example, in the S-S and S-R compatibility literature, congruency conditions usually refers to the congruent and incongruent conditions of a particular ongruency factor.
- I would write" irrelevant stimulus location" instead of "irrelevant Simon location"
- "*the spontaneous decay of response-irrelevant features*" [6]-> this is a bit misleading: stimulus features do not decay. According to Hommel (e.g., Hommel, 1994), it is the representation of these features that

decays, becomes less active .
-"*longer opportunity for the irrelevant location to decay*" -> "longer opportunity for the irrelevant location CODE to decay".

P. 8_-----
-"*According to this account, the correct response is primed not only by the target feature but also by the irrelevant"* ->"According to this account, in the (Simon) congruent- (flanker) congruent conditions, the correct response is not only activated by the target feature, but also primed .by the .. ". Only in this fully congruent condition , indeed, the values of all the features activate the same (correct) response

-"*conveyed the irrelevant flanker color for the flanker condition*". Again, this "flanker condition" is not clear. Maybe "conveyed the irrelevant flanker color that causes the flanker effect"?

- "*because it was assumed to trigger a location congruent or incongruent to the response"* " The "trigger location" bit is not clear. Maybe it is better to claim "*... because the square location may be either congruent or incongruent with the correct-response location*"

P.9_-------
"*trials compared to the corresponding trials mixing incongruent and congruent features*"->"*trials compared to trials mixing incongruent and congruent features*"-

p. 11_________
" *some spatial overlap in the features of the Simon and flanker conditions*" -> this is not clear. What about "some overlap between the stimulus irrelevant position, which causes the Simon effect, and some spatial feature of the flankers"

- *" the irrelevant features for both conditions*" -> better "the irrelevant features causing both the Simon and flanker effect"

"*The Simon condition included horizontal positions and the flanking characters were presented horizontally*"-> "the irrelevant location leading to the Simon effect was the horizontal position of the stimulus display (flankers + target) and the flankers were also presented horizontally". I suggest changing the other examples (e.g., the one from Rey-Mermet & Gade's study) accordingly.

P.:12______________
The way in which the "learning" hypothesis is explained is not clear. The authors refer to it as a bias in the learning of stimulus-response pairings. Why a "bias"? According to this account, the interaction results from the two compatibility effects (flanker and Simon) PLUS the effect of the different numbers of S-R pairings in the four congruency conditions (i.e., the four trial types), which, in turn, results in a different frequency of presentations of such pairings, The interaction thus would result from the summation of compatibility and episodic-memory effects. This should be better explained. The reader can undestand the main point only after having read the whole paragraph (only on p. 13, line 7, the authors start explaining it) I also suggest eliminating the term bias, which makes the reader think of particular tendencies, inclinations, preferences. What about simply "episodic-memory account"?

"*a small stimulus set (i.e., two stimulus features*" -> this use of the term "feature" might be confusing: usually one refers to a stimulus feature as a stimulus dimension/attribute (e.g., one feature is the colour, another feature is the stimulus position). In this case, however, the authors refer to the value of the feature/dimension. Indeed, two stimulus colours are two values of the same stimulus feature/dimension (i.e., color). Accordingly, when the authors speak about "four features" they are not actually referring to 4 different features of the stimulus (e.g., shape, colour, position, size) but to 4 different values (e.g., red, blue, green, yellow) of the same feature (color).
I suggest replacing the term feature with other terms in the manuscript when appropriate, Note that the authors use "features" with the standard meaning on, e.g., p. 15

- I think that the reader might not understand what the authors mean when they claim that "*Thus, the learning of these stimulus-response pairings should be similar across*"

P.:13 _________-

One reason for this finding might be that Rey-Mermet and Gade presented all possible stimulus-response pairings in each trial type (please note that this information was not provided in Akçay and Hazeltine -> That is not clear at all at this point of the manuscript. The authors have not mentioned yet that when there are many (e.g., 4) possible colours/positions there are different frequencies of S-R pairings in the three different categories of trials and they have not explained yet the consequences in terms of learning/strength of episodic memories.

- " *(i.e., 4 congruent target/flanking colors in each response-congruent location*", -> Given that there are four possible locations that are congruent with the correct response, this is confusing. Maybe better: "(i.e, for each of the four congruent pairings of stimulus and response locations, there was one congruent paring of target and flanker colors)" OR "(ie., for each of the four congruent pairings of target and flanker colors, there was one congruent pairing of stimulus and response locations)".

P.17 __________

" *the interaction should be observed when the facilitation effect (i.e., the difference between congruent and neutral trials) was used as dependent measure.* -> The dependent measures were always RTs and errors. Maybe the authors meant that "the interaction should be observed when the analyses focussed on the facilitation effect". The authors use "dependent measure" with the standard meaning in other parts of the manuscript.

P. 48___________

"*Together, the RTs findings of Experiments 1a and 1b emphasized the difficulty of finding appropriate Simon neutral trials on which performance is slower than congruent trials but faster than incongruent trials*" I suggest the authors underline that such a problem concerns only this particular task. Many previous studies found appropriate neutral Simon conditions in standard Simon tasks (e.g., Umiltà, Rubichi, Nicoletti, 1999: Wuhr & Ansorge, 2005; Aisenberg and Henik, 2011; Treccani, Cona, Milanese, Umltà, 2018)

P. 52________

"*Moreover, across the experiments, the delta plots showed an inversed U-curved shape for the Simon congruency effect,...., with further RT slowing, the Simon congruency effect diminished and sometimes reversed. These results are rather in line with Hommel than with Treccani and colleagues . A reason for this might be ...*" In my view, the most plausible reason why Treccani et al. found that the Simon effect increases as the RT increases is that they used an accessory-stimulus Simon task. In this kind of tasks, the irrelevant location that causes the effect is not the position of the target. The interfering information is the position of another object (i.e., the accessory stimulus). This task is thought to be quite different from the standard one. In the accessory-stimulus version of the Simon task, just as happens in other variants of the Simon task, increasing Simon effect functions are usually found. In this case, as in other paradigms in which the spatial meaning of the stimulus is not conveyed by the actual physical position of the target, the irrelevant spatial attribute causing the effect takes time to be coded- indeed, in the accessory stimulus task, participants are forced to analyse the target first, given that it is presented at fixation, and then they move attention to the irrelevant accessory stimulus.
