# Supplementary material for: Multiplicative priming of the correct response can explain the interaction between Simon and flanker congruency
Source: PLoS One. 2021 Mar 9;16(3):e0248172. doi: 10.1371/journal.pone.0248172 (PMC7943002; doi:10.1371/journal.pone.0248172)
Supplement: Attachment — Submitted filename: Review.docx [file pone.0248172.s003.docx]

The present work aims for a better understanding of the origin of the under-additive interaction between Simon and flanker congruency effect evidenced by previous work. Experiment 1 tests whether the interaction results from a spatial feature overlap between tasks by orthogonally manipulating the spatial configuration between both tasks (Simon horizontal/flanker vertical and Simon vertical/flanker horizontal). A robust interaction between Simon and flanker congruency effects was observed in the two groups, ruling out the spatial overlap hypothesis. Experiment 2 tests whether the interaction stems from a bias in the learning of the stimulus-response mappings. The interaction was still observed when equating the number of stimulus exemplars across all trial types, ruling out this hypothesis. Finally, Experiments 3a and 3b evaluate the hypothesis of a multiplicative priming account. In congruent trials, the correct response is activated by the irrelevant stimulus attribute of each task (the position on the screen and the flankers), which may result in multiplicative priming of the correct response (each irrelevant feature would increase response activation proportional to its activation, which would accelerate processing in congruent-congruent trials). This hypothesis was evaluated by incorporating neutral Simon and flanker trials. If the under-additive interaction results from multiplicative priming induced when the trial is both Simon and flanker congruent, the interaction should be observed when the facilitation effect (neutral minus congruent) is used as the dependent variable, but not when the interference effect (incongruent minus neutral) is used as the dependent variable. According to the authors, the results provide evidence for the multiplicative priming account.

The paper addresses a complex and interesting topic in the field of cognitive psychology. It is well written, and the experiments are rigorously conducted. I enjoyed the thorough theoretical approach to the problem, and found the results from Experiments 1 and 2 compelling. However, I believe that some important methodological and theoretical issues regarding the evaluation of the multiplicative priming account should be addressed before the paper is published.

Major

1- Evaluating the multiplicative priming account requires a valid neutral condition for both Simon and flanker tasks. However, finding an appropriate neutral condition in these tasks has proven difficult, and has been the object of several studies. I was disappointed that these studies were not carefully reviewed in the present work, and some methodological features of Experiments 3a and 3b are potentially problematic.

In the flanker task, the authors use flanker stimuli that are neutral with respect to the response set. The issue here is that these neutral flanker stimuli can still share similarities with members of the stimulus set. This issue has been thoroughly investigated by Charles Eriksen and his colleagues (e.g., Yeh & Eriksen, 1984; Eriksen & St. James, 1986). Specifically, the authors used the colors red, brown, violet, and white as part of the stimulus set, and the colors dark pink or green for their neutral flankers. What evidence do we have that these ‘neutral’ colors do not share any similarity with members of the stimulus set? Dark pink could prime the response associated with violet (or red).

For the Simon task, the authors used two types of neutral trials: stimuli presented at the center of the screen (Exp 3a) and stimuli presented above and below the center of the screen (Exp 3b). Presenting stimuli in the center of the screen gives a processing advantage compared to stimuli presented laterally (because the attentional focus does not need to be shifted), which is what happened in Exp 3a, resulting in a reversed facilitation effect. Exp 3b does not fully control for this issue because the spatial distance between neutral stimuli and the center of the screen is twice smaller than the spatial distance between the center of the screen and the outer lateral stimuli. In this respect, what was the rationale for using four stimulus locations (outer left, inner left, inner right, outer right) in the experiments? This design makes it impossible to have neutral and congruent/incongruent Simon trials presented at equidistance from the screen center.

2- The theoretical foundations of the multiplicative priming hypothesis appear a bit weak. I do not see why facilitation effects in Simon and flanker tasks would interact. The authors mention the Treccani et al (2008) study as support for this multiplicative priming account, but I do not understand why. More problematic, the multiplicative priming hypothesis requires that the irrelevant stimulus attribute of each task generates facilitation in congruent trials. In the Simon task, although the bulk of studies generally found facilitation effects, some studies failed to observe facilitation, and attributed the Simon effect to interference only (Craft & Simon, 1970; Simon & Small, 1969). The picture is even less clear in the flanker task, where many studies incorporating neutral trials failed to find facilitation effects (e.g., Wild Wald et al., 2008; de Bruin & Della Salla, 2018), even when neutral flankers shared feature similarities with the target (Eriksen & Eriksen, 1974; Yeh & Eriksen, 1984; Eriksen & St. James, 1986). This literature must be considered by the authors.

3- Another theoretical issue associated with the multiplicative priming hypothesis concerns the relative time-course of automatic response activations in Simon and flanker tasks. A large body of empirical and computational evidence (see Ulrich’s diffusion model for conflict tasks) that the location-based response activation in the Simon task is much faster than the flanker-based response activation. One may thus wonder how multiplicative priming can occur in this context, because the location-based response activation would have almost fully decayed before the flanker-based activation reaches the response activation stage. The authors provide a tentative explanation in the discussion section but I do not understand it. Could you please clarify?

4- I noticed substantial variations in the evidence in favor of the interaction between Simon and flanker congruency effects on mean RTs between the first three experiments (Exp1: BF_10_ = 3.86; Exp2: 19.38; Exp 3a: 1.65). More problematic, Exp 3b reported weak evidence *against* the interaction model, though an interaction was found on error rates. These variations across experiments are puzzling in light of the minor methodological changes between experiments, and require some explanations. One possibility is that the experiments are underpowered. The authors performed a power analysis and concluded that the minimum sample size to observe the interaction of interest was 9. I do not understand how the minimum sample size for a mixed design ANOVA could be 9 given an effect size of $\eta_{p}^{2}=$.42 and a power of .99. Could you please clarify and specify the value of each parameter in G*Power? In addition, could you please clarify what this minimum sample size represent (minimum sample size for the whole experiment versus minimal sample size for each group)? Finally, the effect sizes for the interaction reported in the present study appear much smaller than the benchmark .42 partial eta-square value, though the use of different effect size statistics ($\eta_{p}^{2}$ versus $\eta_{g}^{2})$ prevents a direct comparison.

Minor

5- The authors consider the possibility of multiple priming of the correct response in congruent-congruent trials. One may wonder if multiple priming of the incorrect response can occur as well, and if this phenomenon could contribute to explain the underadditive interaction.

6-Table S1a: the authors report non-significant Simon and flanker effects for the pure Simon and flanker blocks. I hope it is simply a typo…

7-Analyses for the pure blocks of Experiment 2 are missing.

8-Which error term was used for the follow-up t-tests analyses?

9- The authors should provide the mapping between each key and the corresponding hand/finger.

10- It looks like the color-response mapping was fixed across participants for each experiment. What was the motivation for fixing this mapping?

11- Please check the syntax of the sentence lines 617-620.

12- I feel like the hypothesis of a bias in the learning of the stimulus-response mappings (and the way the authors tested for this hypothesis in Experiment 2) will be hard to understand for somebody who is not expert in the field. I may be useful to detail this hypothesis a bit more, perhaps with an illustration. This illustration could also include the other hypotheses (spatial overlap and multiplicative priming).
