# Supplementary material for: Multiplicative priming of the correct response can explain the interaction between Simon and flanker congruency
Source: PLoS One. 2021 Mar 9;16(3):e0248172. doi: 10.1371/journal.pone.0248172 (PMC7943002; doi:10.1371/journal.pone.0248172)
Supplement: Attachment — Submitted filename: Response To Reviewers.docx [file pone.0248172.s004.docx]

# Editor

# *E1 “Please ensure that your manuscript meets PLOS ONE's style requirements, including those for file naming.”*

RESPONSE: We checked our manuscript so that it meets PLOS ONE’s style requirements.

# *E2 “Thank you for including your ethics statement: "The study was conducted in accordance with the guidelines of the ethics committee of the Catholic University of Eichstätt-Ingolstadt (approval number: 2016/18), and written informed consent was acquired from all participants.".*

***a) Please amend your current ethics statement to confirm that your named institutional review board or ethics committee specifically approved this study.***

***b) Once you have amended this/these statement(s) in the Methods section of the manuscript, please add the same text to the “Ethics Statement” field of the submission form (via “Edit Submission”)”***

RESPONSE: In the revised manuscript, we rewrote the ethics statement as follows (see p. 18): “The study was approved by the ethics committee of the Catholic University of Eichstätt-Ingolstadt (approval number: 2016/18), and written informed consent was acquired from all participants”. We also added this text to the “Ethics Statement” field of the submission form.

# Reviewer A

## *A1 “Evaluating the multiplicative priming account requires a valid neutral condition for both Simon and flanker tasks. However, finding an appropriate neutral condition in these tasks has proven difficult, and has been the object of several studies. I was disappointed that these studies were not carefully reviewed in the present work, and some methodological features of Experiments 3a and 3b are potentially problematic.*

***In the flanker task, the authors use flanker stimuli that are neutral with respect to the response set.*** ***The issue here is that these neutral flanker stimuli can still share similarities with members of the stimulus set. This issue has been thoroughly investigated by Charles Eriksen and his colleagues (e.g., Yeh & Eriksen, 1984; Eriksen & St. James, 1986). Specifically, the authors used the colors red, brown, violet, and white as part of the stimulus set, and the colors dark pink or green for their neutral flankers. What evidence do we have that these ‘neutral’ colors do not share any similarity with members of the stimulus set? Dark pink could prime the response associated with violet (or red).***

***For the Simon task, the authors used two types of neutral trials: stimuli presented at the center of the screen (Exp 3a) and stimuli presented above and below the center of the screen (Exp 3b). Presenting stimuli in the center of the screen gives a processing advantage compared to stimuli presented laterally (because the attentional focus does not need to be shifted), which is what happened in Exp 3a, resulting in a reversed facilitation effect. Exp 3b does not fully control for this issue because the spatial distance between neutral stimuli and the center of the screen is twice smaller than the spatial distance between the center of the screen and the outer lateral stimuli. In this respect, what was the rationale for using four stimulus locations (outer left, inner left, inner right, outer right) in the experiments? This design makes it impossible to have neutral and congruent/incongruent Simon trials presented at equidistance from the screen center.”***

RESPONSE: In the present study, in particular in Experiment 1 (previously Experiment 3), we aimed to focus on the impact of response conflict. The reason is that incongruent trials are mainly assumed to induce a response conflict (in particular under no task-switching conditions, such as in the present study; Steinhauser & Hübner, 2009). Accordingly, we defined a trial as neutral when it includes only one response-relevant feature, such as flanker neutral trials in which the flankers were presented in color associated to none of the responses. Nevertheless, we agree with Reviewer A that the impact of similarity in stimulus set and thus the resulting stimulus conflict might be the topic of future work. This is acknowledged in the manuscript (see p. 52-53).

In the present study, we opted for a Simon condition with four stimulus locations because the research using a large stimulus set has resulted in mixed evidence (see Table 1). Moreover, in Experiment 1b (previously Experiment 3b), we did not select a Simon condition with four neutral locations (i.e., outer above, inner above, inner below, outer below) because this would have resulted in an unbalanced number of stimulus-response pairings for Simon neutral trials in comparison to flanker neutral trials (2 stimulus-response pairings). Thus, with our design, we avoided stimulus-response pairings of flanker neutral trials to be presented more frequently and to be learned more efficiently because of their smaller number. This is now clarified in the manuscript (see p. 16).

***A2 “The theoretical foundations of the multiplicative priming hypothesis appear a bit weak. I do not see why facilitation effects in Simon and flanker tasks would interact. The authors mention the Treccani et al (2008) study as support for this multiplicative priming account, but I do not understand why. More problematic,*** ***the multiplicative priming hypothesis requires that the irrelevant stimulus attribute of each task generates facilitation in congruent trials. In the Simon task, although the bulk of studies generally found facilitation effects, some studies failed to observe facilitation, and attributed the Simon effect to interference only (Craft & Simon, 1970; Simon & Small, 1969). The picture is even less clear in the flanker task, where many studies incorporating neutral trials failed to find facilitation effects (e.g., Wild Wald et al., 2008; de Bruin & Della Salla, 2018), even when neutral flankers shared feature similarities with the target (Eriksen & Eriksen, 1974; Yeh & Eriksen, 1984; Eriksen & St. James, 1986). This literature must be considered by the authors”***

RESPONSE: In the manuscript (see p. 8-9), we have now rewritten the paragraph explaining how Treccani et al. (2008) support the multiplicative priming account: “First empirical support for such a priming of irrelevant features was provided by Treccani and colleagues (2009). In their study, both Simon and flanker conditions were so combined that a colored square was presented in the middle of the screen, which was flanked by one colored square either to the left or to the right (see Fig 1). Thus, the flanker square conveyed the irrelevant flanker color for the flanker condition. At the same time, it also conveyed the irrelevant location for the Simon condition because it was assumed to trigger a location congruent or incongruent to the response location. Thus, in congruent-congruent trials, the irrelevant square triggers the correct response twice (i.e., by means of its color and its position; see the top-left panel of Fig 1), thus facilitating performance. Moreover, in this particular design, the irrelevant square also triggers the incorrect response in incongruent-incongruent trials twice (again by means of its color and its position; see the bottom-right panel of Fig 1). This allows a faster rejection of this response. Together, this accounts for the interaction between Simon and flanker congruency by explaining the faster and more correct response in congruent-congruent trials and incongruent-incongruent trials compared to the corresponding trials mixing incongruent and congruent features.”

Moreover, we thank Reviewer A for putting our attention to the literature questioning whether irrelevant stimulus feature can generate facilitation in congruent trials. We have now integrated them in the following way (see p. 17): “However, the multiplicative priming account is based on the assumption that the irrelevant stimulus feature for each Simon and flanker condition co-activate the correct response in congruent trials. As there has been some debate in previous research whether this occurs (de Bruin & Sala, 2018; B. A. Eriksen & Eriksen, 1974; C. W. Eriksen & St. James, 1986; Simon & Craft, 1970; Simon & Small, 1969; Wild-Wall et al., 2008; Yeh & Eriksen, 1984), it is possible that both the irrelevant location and flanking letters do not co-activate the correct response. In this case, the interaction should not be observed in the facilitation effect. This implies that the interaction should be observed in the interference effect. The reason is that as the congruency effect is computed as the addition of the interference effect and the facilitation effect, finding an interaction in the congruency effect but not in the facilitation effect should result in finding an interaction in the interference effect.”

***A3 “Another theoretical issue associated with the multiplicative priming hypothesis concerns the relative time-course of automatic response activations in Simon and flanker tasks. A large body of empirical and computational evidence (see Ulrich’s diffusion model for conflict tasks) that the location-based response activation in the Simon task is much faster than the flanker-based response activation. One may thus wonder how multiplicative priming can occur in this context, because the location-based response activation would have almost fully decayed before the flanker-based activation reaches the response activation stage. The authors provide a tentative explanation in the discussion section but I do not understand it. Could you please clarify?”***

RESPONSE: In addition to mentioning Ulrich’s diffusion modeling approach (Ellinghaus et al., 2018; Ulrich et al., 2015), we clarified our explanation by illustrating it with a new figure (i.e., see p. 50-51 and Fig 10).

***A4 “I noticed substantial variations in the evidence in favor of the interaction between Simon and flanker congruency effects on mean RTs between the first three experiments (Exp1: BF10 = 3.86; Exp2: 19.38; Exp 3a: 1.65). More problematic, Exp 3b reported weak evidence against the interaction model, though an interaction was found on error rates. These variations across experiments are puzzling in light of the minor methodological changes between experiments, and require some explanations. One possibility is that the experiments are underpowered. The authors performed a power analysis and concluded that the minimum sample size to observe the interaction of interest was 9. I do not understand how the minimum sample size for a mixed design ANOVA could be 9 given an effect size of*** $\boldsymbol{\eta}_{\boldsymbol{p}}^{\boldsymbol{2}}\boldsymbol{=}$***.42 and a power of .99. Could you please clarify and specify the value of each parameter in G*Power? In addition, could you please clarify what this minimum sample size represent (minimum sample size for the whole experiment versus minimal sample size for each group)? Finally, the effect sizes for the interaction reported in the present study appear much smaller than the benchmark .42 partial eta-square value, though the use of different effect size statistics (***$\boldsymbol{\eta}_{\boldsymbol{p}}^{\boldsymbol{2}}$ ***versus*** $\boldsymbol{\eta}_{\boldsymbol{g}}^{\boldsymbol{2}}\boldsymbol{)}$ ***prevents a direct comparison”***

RESPONSE: The variation in Bayes factors (BF) across the experiments might be caused by differences in the number of participants, the number of trials, and the number of repetition of stimulus-response pairings per trial type.

| Experiment | Number of participants | Number of trials in the mixed blocks per participant and trial type | Number of repetition of stimulus-response pairings per participant and per trial type |
| --- | --- | --- | --- |
| 1a (previously 3a) | 29 | 160 | Congruent-congruent trials: 40  Trials mixing incongruent and congruent stimuli: 13-14  Trials mixing incongruent and neutral stimuli: 53-54  Trials mixing neutral and congruent stimuli: 40  Neutral-neutral trials: 20  Incongruent-incongruent trials: 4-5 |
| 1b (previously 3b) | 30 | 160 | Congruent-congruent trials: 40  Trials mixing incongruent and congruent stimuli: 13-14  Trials mixing incongruent and neutral stimuli: 26-27  Trials mixing neutral and congruent stimuli: 20  Neutral-neutral trials: 10  Incongruent-incongruent trials: 4-5 |
| 2 (previously 1) | 48 (i.e., 24 per group) | 216 | Congruent-congruent trials: 54  Trials mixing incongruent and congruent stimuli: 18  Incongruent-incongruent trials: 6 |
| 3 (previously 2) | 36 | 216 | 54 |

Thus, the larger BF observed in Experiment 3 (previously Experiment 2) might result from a certain consistency observed in the RT data due to the larger sample size and the large number of repetitions of each stimulus-response pairing for each trial type.

Regarding the BF = 21.53 observed in error rates for the interaction between Simon and flanker conditions in the Experiment 1b (previously Experiment 3b), this seems to be associated to a slightly smaller range in the mean error rates in this experiment (i.e., CIs = 5%-8% compared to 5%-9% in Experiment 1a, for example). At the same time, the interaction – represented as the difference in the flanker congruency effect between Simon incongruent and congruent trials – is slightly larger in Experiment 1b (i.e., 3% in comparison to 2% in Experiment 1a). It is possible that both patterns – that is, smaller error-rates range but larger difference – explains this BF.

In addition, we would like to thank Reviewer A for pointing out our error regarding the power analysis. We removed the power analysis because we misread G*Power requirements, which seems to occur very often (see Brysbaert, 2019). We also refrained us to compute a post-hoc power analysis because this is not appropriate (see <http://daniellakens.blogspot.com/2014/12/observed-power-and-what-to-do-if-your.html>). Moreover, if our experiments are underpowered, this means that all previous studies investigating the interaction between Simon and flanker conditions were underpowered. The reason is that we selected our minimal sample size (i.e., 24 participants in each group in which the interaction between Simon and flanker conditions was expected to occur) because it belongs the largest sample sizes tested in previous research (see Table 1).

**MINOR POINTS**

1. ***“The authors consider the possibility of multiple priming of the correct response in congruent-congruent trials. One may wonder if multiple priming of the incorrect response can occur as well, and if this phenomenon could contribute to explain the underadditive interaction.”***

RESPONSE: Yes, the multiplicative priming was also used to explain performance in incongruent-incongruent trials. This is now clarified in the manuscript (see p. 8-9): “Moreover, in [the] particular design [used by Treccani and colleagues (2009)], the irrelevant square also triggers the incorrect response in incongruent-incongruent trials twice (again by means of its color and its position; see the bottom-right panel of Fig 1). This allows a faster rejection of this response. Together, this accounts for the interaction between Simon and flanker congruency by explaining the faster and more correct response in congruent-congruent trials and incongruent-incongruent trials compared to the corresponding trials mixing incongruent and congruent features.”

2. ***“Table S1a: the authors report non-significant Simon and flanker effects for the pure Simon and flanker blocks. I hope it is simply a typo…”***

RESPONSE: Yes, it was a typo. Thank you for pointing out this error. This has been changed (see Table S1c in the S1 file on p. 4-5).

3. ***“Analyses for the pure blocks of Experiment 2 are missing.”***

RESPONSE: These analyses for Experiment 3 (previously Experiment 2) are presented in Table S1d in the S1file (see p. 6 in the S1 file).

4. ***“Which error term was used for the follow-up t-tests analyses?”***

RESPONSE: We used the pooled estimate for the error term.

5. ***“The authors should provide the mapping between each key and the corresponding hand/finger.”***

RESPONSE: This has been now added (see p. 21 and 37).

6. ***“It looks like the color-response mapping was fixed across participants for each experiment. What was the motivation for fixing this mapping?”***

RESPONSE: We followed previous research (Akçay & Hazeltine, 2011; Rey-Mermet & Gade, 2016; Stoffels & Molen, 1988).

7. ***“Please check the syntax of the sentence lines 617-620.”***

RESPONSE: This has been modified (see p. 24): “In the present experiments, the most relevant results are the interaction between both congruency variable, that is, the modulation of the congruency effect (i.e., incongruent vs. congruent trials), the interference effect (i.e., incongruent vs. neutral trials), and the facilitation effect (i.e., neutral vs. congruent trials) of one condition by the congruency of the other condition.”

8. ***“I feel like the hypothesis of a bias in the learning of the stimulus-response mappings (and the way the authors tested for this hypothesis in Experiment 2) will be hard to understand for somebody who is not expert in the field. I may be useful to detail this hypothesis a bit more, perhaps with an illustration. This illustration could also include the other hypotheses (spatial overlap and multiplicative priming).”***

RESPONSE: As suggested by Reviewer A, we added figures to explain the learning of stimulus-response pairings (see Fig 2) and the multiplicative priming effect (see Figs 1 and 3).

# Reviewer B

## *B1 “Results of this study are interesting but the way in which they are presented is not convincing. There are many logic inconsistencies and the rationale of the experiments is weak: the proposed accounts cannot explain the critical interaction and/or the proposed experiment is not appropriate to evaluate the account. I suggest starting from the multiplicative priming account, which appears to be the most sensible, sound and grounded account based on previous studies, and presenting feature-overlap and frequency of S-R parings as factors they may have an impact on the interaction, i.e., they may be necessary for the multiplicative priming and, consequently, the interaction to occur (feature-overlap) or they may either act in favour or counteract the under-additive interaction predicted by the multiplicative priming account. The study may be presented as an attempt to evaluate the impact of these factors.”*

RESPONSE: We followed Reviewer’s B suggestion, and we re-organized the manuscript accordingly. Thus, Experiments 3a and 3b are now Experiments 1a and 1b, respectively, and Experiments 1 and 2 are now Experiments 2 and 3, respectively. We also present all these experiments as testing whether the interaction between Simon and flanker congruency is affected by the multiplicative priming of the correct response, the spatial overlap between Simon and flanker features, and the bias in the learning of stimulus-response pairings (see p. 2, 14-15, 35-36, 41-42, and 47-48).

## *B2.1 “Other accounts of the interaction (conflict-monitoring and spatial-code decay). p.8. lines 117-125. I do not fully understand why the results of the ERP study described here rule out the conflict-monitor and spatial-code decay accounts. Please, give a thorough and clear explanation for that.“*

RESPONSE: We now explained in more details the results of the ERP study and how they challenge both accounts (see p. 10): “Second, the central assumption put forward in both accounts – that is, resolving the first conflict affects the resolution of the second conflict – has been put into question by a study using event-related potentials (ERPs; Frühholz et al., 2011; see also Rey-Mermet et al., 2019). In that study, ERPs were used to track the neural time course of conflict resolution. The results showed an interaction in RTs, but a sequential conflict resolution in the ERPs. That is, the resolution of the flanker conflict was associated with an early component (i.e., N2), whereas the resolution of the Simon conflict was associated with a later component (i.e., P3b). However, no interaction was observed in the ERPs, in particular on the later component. If the first conflict affected the resolution of the second conflict, this should have resulted in an interaction in the later component because this later component is associated to the resolution of the second conflict. In contrast, the results suggest that although flanker conflict and Simon conflict are resolved at separable stages, there appears to be little influence of the earlier stage on the later one.”

## *B2.2 “Beyond this, the results of ONE experiment are not enough to rule out an account - especially because: (a) as I understand it, the critical results challenging that account are ONLY the ERP data of that study ; (b) the conflict-monitoring account is presented in the paper as one of the main accounts for the interaction. I do not actually think that the conflict-monitoring account is the main account for the interaction. In my view it is a sensible explanation for sequential effects, but it is not a sound explanation for this type of interaction (see my second minor point).*

***In any case, among the studies that the authors mention, there may be other studies the results of which challenge these two accounts (conflict-monitor and spatial-code decay). For example, did other studies perform distributional analyses? Did other studies find additive effects instead of interactive effects? Did other studies find other types of interactions? Are these additive effects/other types of interactions accountable for by these two hypotheses?
From what I understand, the results of the present study do not rule out these alternative accounts (both accounts are compatible with the results), thus the authors should present convincing evidence that the multiplicative priming account is the most suitable explanation for the interaction.”***

RESPONSE: Our goal was not to say that the within-trial conflict-monitoring and spatial-code decay accounts are ruled out. Rather, our goal was – for the sake of completeness – to report the accounts used in previous research to explain the interaction of this kind, and then to highlight the findings which are not line with these accounts. Moreover, in case of the multiplicative-priming account, our goal was to highlight the fact that this account seems very appropriate to explain the findings when all irrelevant features are conveyed by the same object, but it is unknown whether this is still the case when a more standard design is used (i.e., when irrelevant features are conveyed by different objects).

Overall, we think that these accounts can so far explain some but not all empirical results. Accordingly, more than one process could account for the under-additive interaction between flanker and Simon congruency. This is now clarified in the manuscript (p. 9-11).

## *B3 “On p. 8, the authors claim that “although flanker conflict and Simon conflict are resolved at separable stages, there appears to be little influence of the earlier stage on the later one.” What are these different processing stages? That the flanker and Simon effects arise at different processing stage is indeed inconsistent with: (a) the conclusions of the present study. According to the additive factor logic, the presence of an interaction suggests that two effects arise at the same stage, or involve at least one processing stage in common (cf. Sternberg, 1969, 1984). Moreover, if, as suggested by the authors, the interaction is due to priming of the same vs. different response(s) by the two irrelevant features, then we can conclude that both effects emerge at the response-selection stage. (b) main accounts of the flanker and Simon effects, according to which they both emerge at the response selection stage. There are plenty of evidence suggesting that the locus of both effects is that stage (cf., e.g., literature reviews by Proctor and colleagues, Eriksen and colleagues, experimental studies like that by Hübnerand Töbel, 2019) and I think that the authors should explicitly state it. They should also underline that, given that both effects arise at the same processing sage, one may expect an interaction between the two compatibility factors.”*

RESPONSE: Reviewer B exactly points out the issue in previous research. According to the additive factor logic, the interaction in RTs observed between Simon and flanker congruency should occur because two variables influence the same processing stage (most probably the response selection or execution stage; see Steinhauser & Hübner, 2009). At the same time, when the Simon and flanker tasks were investigated separately (e.g., Ellinghaus et al., 2018; Gade et al., 2020; Hübner & Töbel, 2019; Ulrich et al., 2015; Vallesi et al., 2005) or combined together (Frühholz et al., 2011; Hommel, 1997), the results showed different temporal resolution for flanker and Simon tasks. How can we reconcile both aspects? In a tentative way, we proposed that the interaction does not emerge during conflict resolution, but appears on the level of motor responses. For example, the flankers and the irrelevant location could activate their respective motor responses via direct associations already during early stimulus processing (see Hübner et al., 2010). Thus, the flanker condition is first processed, pre-activating the correct response. Then, the Simon condition is processed, pre-activating the correct response again. These pre-activations of motor responses then result in a co-activation of the correct response at the stage of response selection or execution, which affects the duration of response selection or response execution, and in consequence performance. Please note that in addition to mentioning the additive factor logic in the revised version of the manuscript (see p. 50-51), we added a figure (Fig 10) to illustrate this explanation.

## *B4.1 “The feature overlap account is NOT an account for the interaction, but only a possible account for the discrepancies between studies: according to this account, the interaction can only emerge when there is an overlap between the irrelevant spatial feature producing the Simon effect and a spatial feature of the flanker object(s), thus this overlap is a prerequisite for the interaction (and this would explain why in some studies no interaction emerged while it emerged in others), but it does not specify which is the mechanism producing the interaction. Accordingly, feature overlap should not be presented as an account for the interaction, but as a possible factor influencing it.”*

RESPONSE: We agree with Reviewer B, and we now present spatial feature overlap as a possible factor influencing the interaction between Simon and flanker congruency (see p. 11-12).

## *B4.2 “It is worth noticing that a paper mentioned by the authors on p. 5 and p. 45 (Treccani et al., 2012) proposes that the interaction only emerges when there is massive overlap between the two irrelevant features (i.e., they are borne by the very same object) and participants process such an overlap (i.e., they “bind” the two features). Thus the “feature binding” is the prerequisite. Yet, the mechanism proposed to account for the interaction is multiplicative priming.”*

RESPONSE: We now better highlight that it is unknown whether the multiplicative priming account can still explain the interaction between Simon and flanker congruency when a more standard design is used (i.e., when irrelevant features are conveyed by different objects). We did it as follows (see p. 10-11): “The multiplicative priming effect of the correct response has been so far tested only by Treccani and colleagues (2009, 2012). In comparison to previous studies (see Table 1), they used a very particular design in which the same object – that is, the irrelevant square – conveyed the irrelevant features for both conditions. This may have changed the weight for processing the irrelevant features so that the irrelevant features could trigger the associated response. Thus, it is possible that in all other studies reported in Table 1 in which the Simon and flanker conditions were combined in a more standard way – that is, with different objects conveying the irrelevant features for both conditions – the priming effect cannot account for the interaction between Simon and flanker congruency. This might be particularly the case because there is some debate in previous research whether in standard Simon and flanker tasks, the irrelevant stimulus feature can co-activate the correct response in congruent trials at all (de Bruin & Sala, 2018; B. A. Eriksen & Eriksen, 1974; C. W. Eriksen & St. James, 1986; Simon & Craft, 1970; Simon & Small, 1969; Wild-Wall et al., 2008; Yeh & Eriksen, 1984).”

## *B5 “pp. 9-11. The account based on the “learning of stimulus-response mappings” does not actually account for the critical interaction, at least as it is presented (see REASONS** below). The authors may consider presenting the frequency of presentation of the different S-R pairings simply as a factor that may influence the performance in this kind of tasks (i.e., tasks combining more than one compatibility factor). They may present Experiment 2 as an experiment aimed to investigate the possible impact of this factor and its possible contribution to the interaction (together with OTHER factors) - in principle, at least, this factor may actually counteract, rather than act in favour of the interaction (see below). If they decide to do so, they should specify in which previous studies this factor may have had an effect, that is, in which previous studies using flanker-Simon paradigms the S-R pairings were presented with a different frequency. Indeed, the rationale of Experiment 2 would become even weaker if in other previous studies the frequencies of different S-R parings were balanced. To make a significant contribution to the understanding of the factors influencing performance in this kind of tasks, I strongly suggest comparing (by means of an ANOVA) the results of Experiment 2 with that of the similar condition of Experiment 1 in which, however, frequencies were not balanced. In this way, the authors may evaluate the specific contribution of this factor. **REASONS WHY THIS ACCOUNT DOES NOT WORK: (a) The authors claim that “the stimulus-response mappings for the congruent-congruent trials are the easiest to learn and then to retrieve because there are only two mappings” This does not make any sense to me : the congruent-congruent trials are not presented in isolation but with the other trials, thus each of these two (I would say) S-R “doubly” congruent pairings are simply one of 8 possible S-R pairings. They cannot be easier to learn or retrieve. (b) The authors probably meant something else: They probably meant that the numbers of times with which the S-R pairings are presented can explain the interaction (i.e., some S-R pairings may be practiced more). However, I do not understand how. - When the stimulus irrelevant features are two (e.g., two positions, two flanker identities), and the study provides for the same number of trials in all the four possible categories (a. flanker congruent-Simon congruent; b. flanker incongruent-Simon incongruent, c. flanker congruent-Simon incongruent; d. flanker incongruent-Simon congruent), the number of S-R pairings for each category is exactly the same (i.e, two S-R pairings for each category), thus one would not expect any interaction. To produce the interaction, a study should provide for a larger number of S-R pairings for the categories a and b than for the categories c and d. For example, a study that considers only three categories: totally congruent trials, totally incongruent trials, partially (either flanker or Simon) incongruent trials. This last category would include more S-R pairings (i.e, 4 exemplars), that would be presented less frequently To the best of my knowledge, a similar study has not been conducted, thus this is not an account for the interaction for any two-feature study (If –instead- I am wrong, the authors should explicitly mention this study and explain that in this specific case the interaction may emerge from the addition of two compatibilities effect and the effect of the number of exemplars/frequency) - When the stimulus irrelevant features are four, and the study provides for the same number of trials in all the four possible categories, the number of S-R pairings for each category is as proposed by the authors: 4 exemplars of the category a (congruent )< 12 exemplars of category c= 12 exemplars of category d< 36 exemplars of category b . The effect of this should be: RT (and/or errors) of aThere is no apparent reason why there should be advantage for the 4-exemplars “a” category over the 12 -exemplars categories, while there should not be an advantage of the 12-exemplars categories (“c” or “d”) over the 36-exemplars category. Quite the opposite, these different frequencies may counteract a possible under-additive interaction produced by other factors (e.g., multiplicative priming). The comparison I suggested (see above) may help to understand the real impact of this factor. (c) The authors also state that “Explaining performance on this trial type may be sufficient to account for the interaction …The reason is that although previous research has focused on the reduction of the congruency effect of one task when the other task was incongruent, the interaction between Simon and flanker congruency mainly resulted from an increase in the congruency effect of one task when the other task was congruent, in particular from the fast RTs and high rates of correct responses in the congruent-congruent trials” As explained above, this pattern of performance cannot be explained by the different frequency of S-R pairings given that, based on that, one should expect larger effects of a compatibility factor in both levels of the other factor. Moreover, I do not think that the interaction mainly results from an increase of the effect of a congruency factor in the congruent level of the other factor: in some studies a congruency effect was found in the congruent level of the other factor, while this effect was no longer significant in the incongruent level of the other factor (thus, a reduction/disappearance of the effect was observed in the other-factor incongruent conditions compared to standard conditions). The authors themselves found a disappearance of the effect of a compatibility factor in the incongruent level of the other factor. E.g.,: “Similarly, the Simon congruency effect was smaller – but not significant – when the trials were flanker incongruent””*

RESPONSE: We followed Reviewer’s B suggestion, and we presented Experiment 3 (previously Experiment 2) so that its goal was to determine whether a bias in the learning of stimulus-response pairings may explain the discrepancy in previous results (see p. 12-14):

“A second issue is the question whether the interaction is affected by episodic memory effects, in particular a bias in the learning of stimulus-response pairings (Dreisbach et al., 2007). In all studies using a small stimulus set (i.e., two stimulus features for each Simon and flanker condition), the interaction was observed (see Table 1). However, in this case, the number of stimulus-response pairings is similar across all trial types (i.e., flanker congruent – Simon congruent, flanker incongruent – Simon congruent, flanker congruent – Simon incongruent, flanker incongruent – Simon incongruent, see Fig 2). Thus, the learning of these stimulus-response pairings should be similar across all trial types.

In contrast, in studies using a larger stimulus set, the results are mixed (see Table 1). Whereas Akçay and Hazeltine (2011) reported no interaction between Simon and flanker congruency, Rey-Mermet and Gade (2016) did observe an interaction. One reason for this finding might be that Rey-Mermet and Gade presented all possible stimulus-response pairings in each trial type (please note that this information was not provided in Akçay & Hazeltine, 2011). However, when the stimulus set consists of four stimulus features (i.e., four locations for the Simon condition and four colors for the flanker condition), the number of stimulus-response pairings differed across trial types (see Fig 2). There are four stimulus-response pairings when both conditions are congruent (i.e., 4 congruent target/flanking colors in each response-congruent location), twelve stimulus-response pairings when one condition is congruent and the other is incongruent (i.e., 4 congruent target/flanking colors x 3 incongruent locations, or 3 incongruent target/flanking colors x 4 congruent locations), and thirty-six stimulus-response pairings when both conditions are incongruent (i.e., 4 target colors x 3 flanking colors x 3 locations). Thus, presenting equally often each trial type (i.e., congruent – congruent, congruent – incongruent, incongruent – congruent, and incongruent – incongruent) results in different frequencies for each stimulus-response pairing. In particular, the stimulus-response pairings from the congruent-congruent trials are presented more often than those of any other trial types. Thus, because this trial type is presented most frequently, there is more chance that participants learn to assign a response to each stimulus exemplar. This may encourage participants to base their performance for this trial type on direct stimulus-response pairings. Assuming that this learning is linear, it is possible that the stimulus-response pairings in trials mixing congruent and incongruent features are better learned than those in incongruent-incongruent trials. The reason is that there are more repetitions of stimulus-response pairings in trials mixing incongruent and congruent trials than those in incongruent-incongruent trials. However, there is no a priori reason to prefer a linear learning process. Therefore, if a bias in the learning of stimulus-response pairings changes the interaction between Simon and flanker congruency, it should be best observed in congruent-congruent trials. This would explain why responses were the fastest and the most correct for the congruent-congruent trial type. Critically, explaining performance on this trial type may be sufficient to account for the interaction between Simon and flanker congruency observed in Rey-Mermet and Gade (2016). In that study, the interaction was driven by fast RTs and high rates of correct responses in the congruent-congruent trials. There was no decrease in RTs (i.e., faster RTs) or increase in correct responses (i.e., higher rates of correct response) in the incongruent-incongruent trials.”

In this context, we also performed an analysis comparing Experiment 3 (previously Experiment 2) with the corresponding condition of Experiment 2 (previously Experiment 1, that is, the group with vertical flankers and horizontal Simon locations). The results revealed that the interaction between Simon and flanker congruency did not differ across both experiments (see p. 46-47).

## *B6 “The rationale of Experiments 3a and 3b is not clear. Do these two experiments simply aim to verify a prediction of the multiplicative priming account? The authors claim that “the interaction should not be found when the interference (i.e., the difference between incongruent and neutral trials) was used as dependent measure”. What are the predictions based on other accounts? Could we expect other outcomes based on the other factors mentioned by the authors?”*

RESPONSE: The goal of Experiments 1a and 1b (previously Experiments 3a and 3b) was to determine whether the under-additive interaction between Simon and flanker congruency results from the multiplicative priming of the correct response in a design in which – contrary to the design used by Treccani and colleagues (2009, 2012) – the irrelevant features for both Simon and flanker conditions are conveyed by different objects (see Fig 3). With such a design, in congruent-congruent trials, the correct response is activated not only by one of the irrelevant features (e.g., the position on the screen) but also by the other irrelevant feature (e.g., the flanker; see the top-left panel of Fig 3). This may result in multiplicative effects of priming of the correct response due to co-activation (Mittelstädt & Miller, 2018; Treccani et al., 2009). Thus, this would accelerate trial processing for the congruent-congruent trials not only compared to when the response is primed by only one irrelevant feature (see all other panels of Fig 3), but its facilitative effect would be also stronger than the sum of the single priming effects. This over-additive priming would result in an under-additive RT pattern because the processing of congruent-congruent trials is selectively accelerated.

However, the multiplicative priming account is based on the assumption that the irrelevant stimulus feature for each Simon and flanker condition co-activate the correct response in congruent trials. As there has been some debate in previous research whether this occurs (de Bruin & Sala, 2018; B. A. Eriksen & Eriksen, 1974; C. W. Eriksen & St. James, 1986; Simon & Craft, 1970; Simon & Small, 1969; Wild-Wall et al., 2008; Yeh & Eriksen, 1984), it is possible that both the irrelevant location and flanking letters do not co-activate the correct response. In this case, the interaction should not be observed in the facilitation effect. This implies that the interaction should be observed in the interference effect. The reason is that as the congruency effect is computed as the addition of the interference effect and the facilitation effect, finding an interaction in the congruency effect but not in the facilitation effect should result in finding an interaction in the interference effect. This is now clarified in the manuscript (see p. 15-17).

**MINOR POINTS**

1. ***“From the manuscript: “Simon Task, flanker task, and their interaction” “Previous research has shown an interaction between the Simon and flanker tasks when both tasks were combined within the same trial”.
   It is worth noticing that the interaction is not between two tasks. Indeed, the task is only one: participants had to indicate the colour of the central letter while ignoring the flanking letters and their location on the screen. This task had the characteristics of both typical Simon and flanker tasks.
   I would say that the interaction is between the flanker-target identity congruence (the compatibility factor usually observed in flanker tasks) and the stimulus-response spatial correspondence (the compatibility factor usually observed in flanker tasks).
   Speaking about two tasks may be confusing to the reader (one may think of a dual-task paradigm) and makes it harder to understand the proposed accounts for the interaction.
   For example, that authors claim that “One can assume that the conflict monitoring system estimates the current levels of conflict for the task being processed first. If this task is incongruent and thus associated with a high degree of conflict, this leads to a shift of control signal. That is, response-relevant features are activated while all irrelevant features – including those for the task being processed second – are inhibited. Thus, the strength of control is also adjusted for the second task to be processed. “
   What are the tasks the authors are speaking about?
   I guess they are speaking about irrelevant features, instead of tasks, which are not to be processed (given that they are task-irrelevant), but they are processed nevertheless.
   I would rather write: “One can assume that the conflict monitoring system estimates the current levels of conflict. If the first irrelevant feature being processes (e.g., stimulus position) is incongruent with the relevant one, and thus associated with a high degree of conflict, response-relevant features are activated while all irrelevant features (not only that already processed) are inhibited.“***

RESPONSE: We agree with Reviewer B, and we changed the terminology accordingly throughout the manuscript where appropriate. Moreover, we changed the description of the within-trial conflict monitoring (see p. 7): “Here, one could assume that if the first irrelevant feature being processed (e.g., the irrelevant flanker) is incongruent with the relevant one and thus associated with a high degree of conflict, this leads to a shift of control signal. As a consequence, the response-relevant feature is activated while all irrelevant features – including the irrelevant Simon location – are inhibited. Thus, the strength of control is also adjusted for the second irrelevant feature. This reduces the impact of the irrelevant features if the second irrelevant feature is also incongruent, thus resulting in a smaller congruency effect. According to this account, control processes generalize across all irrelevant features, explaining the under-additive interaction observed in most previous studies.”

2. ***“Also, what is exactly inhibited according to the conflict-monitor account? Following some versions of this account (e.g., the “gate” version), when a conflict between competing response codes is detected by the conflict monitoring mechanism, the weight of the route activated by the irrelevant feature(s) is attenuated. Do the different versions of the conflict-monitoring account have different predictions?”***

RESPONSE: At first glance, we would say that the different versions of this account would not differ in their predictions.

3. ***Actually, as underlined above, I find this account not very convincing. If the gate closes, it should close in the first place for the direct route involving the feature for which a conflict is detected. Moreover, it should take a while for the gate to close, thus the effect of this closure should be observed in the following trial.”***

RESPONSE: This account is presented because previous research have mentioned it as an explanation for such interactions.

4. ***“p.8 " Hommel [6] (see Table 1), suggesting that this effect could explain the observed interaction in some experiments but not in others".
   What effect? Please be more explicit “suggesting that this phenomenon(i.e., the decay of the stimulus spatial code) could explain”***

RESPONSE: We changed the text as follows (see p. 10): “the decay of response codes could explain the observed interaction in some experiments but not in others”.

5. ***“Please fix inconsistencies in lexical choices. “Stimulus-response mapping” (e.g., p.9) usually means the instructed (i.e., contained in the instructions) associations between specific stimulus values and the correct responses(i.e., the responses required by these values). I think that the authors should use instead “S-R pairings” (e.g., S-R congruent pairings). Note that the authors themselves use “stimulus-response mapping” with the standard meaning on p. 14.***

RESPONSE: We thank Reviewer B for proposing such a clear differentiation. We applied it throughout the manuscript.

6. ***p. 14. “The stimulus was determined randomly for each trial” Maybe better (more clear) “the stimulus colour was…”.***

RESPONSE: We changed the sentence according to the suggestion (see p. 18)

7. ***“p. 16. In contrast with the horizontal-key condition, the keys in the vertical-key condition are not perfectly aligned along one spatial axis, the vertical one (i.e., they are not perfectly one above the other). Can the authors justify this choice? (in many keyboards, there are numerical keys which are vertically aligned).”***

RESPONSE: We opted for these keys because they are next to the keys we used for the horizontal Simon condition in this study as well as in the previous one (Rey-Mermet & Gade, 2016). In addition, if we selected keys from the numerical keyboard, we would have had a problem for the horizontal Simon condition. Here, there are no four keys in a row.

8. ***“pp. 14-16. I did not understand whether stimuli were of different size in the different conditions (it is not clear from the description) and whether in the flanker pure blocks the asterisks appeared in a (central) yellow square as in the pure Simon blocks. These seem unnecessary differences between experimental conditions***.***”***

RESPONSE: In the pure Simon and flanker blocks as well as in the mixed blocks, all stimuli were presented in 28-point Arial Bold font. In the pure flanker blocks, the stimuli were not presented in a yellow square because all stimuli were presented centrally. This is now clarified in the manuscript (see p. 19).
