# Supplementary material for: Multiplicative priming of the correct response can explain the interaction between Simon and flanker congruency
Source: PLoS One. 2021 Mar 9;16(3):e0248172. doi: 10.1371/journal.pone.0248172 (PMC7943002; doi:10.1371/journal.pone.0248172)
Supplement: Attachment — Submitted filename: Responses to R2.docx [file pone.0248172.s007.docx]

# Reviewer A

## *A1.1 ““The account presented here is so far very similar to the temporal overlap account proposed by Hommel (1997), except to one key difference. Here, we do not assume a spontaneous decay of the representation features” I think that the account presented here is very different from the account that Hommel proposed in 1997 for the interaction between S-R correspondence and S-S congruency. According to Hommel, S-S congruency (the compatibility between the target and flanker) affects the perceptual encoding of the stimulus, stimulus identification, while S-R correspondence (the correspondence between stimulus and response positions) affects response selection. In S-S incongruent trials, stimulus identification would be delayed and thus, when response section starts, the irrelevant spatial code would not be active anymore (either because of the decay of the spatial code or because of active inhibition of this spatial code - that does not matter in this context). Accordingly, this code would not have any effect on response selection (->under-additive interaction). In contrast, the authors here propose that both S-S congruency and S-R correspondence affect response selection: Both irrelevant features prime the congruent/correspondent response at the response selections stage.* *If there is an overlap in time between the effects of these priming processes (if the response primed by a feature is still activated when the second priming process occurs) we observe the interaction. Thus, in order for the interaction to be observed, the overlap does not need to occur between the processing of the two features,* *but between the effect of the processing of the two features: the effect of the processing of one feature (i.e., the response priming mediated by one feature) must overlap in time with the effect of the processing of the other feature (i.e. the response priming mediated by the other feature).*

## *In this respect, in my view, the two accounts that the authors propose in the ‘Discussion’ section (pp. 53-57) are not that far from each other.”*

RESPONSE: Following Reviewer’s comment, we re-wrote the sentences in order to make clear the similarity (i.e., the assumption of a temporal overlap) and the key difference (i.e., no assumption about which process is responsible for this overlap) between Hommel’s (1997) account and the framework presented in the manuscript. We did it as follows (see p. 54):

“Similar to Hommel [6], a temporal overlap is assumed in this account. However, there is at least one key difference. No spontaneous decay of the representation features is assumed. In this view, whereas the spontaneous decay of the representation features is still an option to explain the interaction between Simon and flanker congruency, the inhibition of irrelevant features (as derived from the conflict monitoring account) or the multiplicative priming of the correct response are also valid options.”

Moreover, the Reviewer suggested similarities between the temporal overlap account presented on p.53-54 and the account assuming the pre-activation of the correct response. These are now reported as follows (see p. 56): “It should be noted that according to this explanation [that is, the pre-activation of motor responses by relevant and irrelevant features], there is still an overlap in time. However, this temporal overlap is not between the processing of the relevant and irrelevant features, such as described above or proposed by Hommel [6]. Rather, it is between the effects of processing the irrelevant features. That is, the interaction between Simon and flanker congruency occurs only if the effect of processing one irrelevant feature (i.e., the response priming mediated by one irrelevant feature) overlaps in time with the effect of processing the other irrelevant feature (i.e. the response priming mediated by the other irrelevant feature).”

## *A2 “In the cover letter the authors claim “ the EEG findings are not directly compatible with an account assuming an overlap in time. The EEG results clearly showed separate ERPs associated to the processing Simon and flanker irrelevant features.” However,* *the lack of an overlap in time between two ERP components reflecting the processing of two stimulus features is totally consistent with an overlap in time of the effects of the processing of these two features: one feature can be processed and its effect (i.e., response activation/priming produced by the processing of this feature) can last over time.”*

RESPONSE: Following Reviewer’s suggestion, we modified the manuscript as follows (see p. 56): “This assumption [that is, the effect of processing one irrelevant feature overlaps in time with the effect of processing the other irrelevant feature] is also compatible with the EEG findings showing separate ERPs associated with the processing of the irrelevant features for both Simon and flanker congruency variables [5, 23,24]. The reason is that the lack of a temporal overlap between the two ERP components reflecting the processing of relevant and irrelevant features is totally consistent with a temporal overlap in the effects of processing these features: One feature can be processed and its effect – that is, the response priming mediated by the processing of this feature – can last over time.”

***A3 “******Finally in the Discussion section the authors claim that: “if one considers that the interaction between Simon and flanker congruency does not emerge during conflict processing, but appears on the level of motor responses.”
This is confusing: According to main models of response selection, in the case of tasks such as Simon and flanker tasks, conflict processing DOES occur at a motor-related stage, namely, response selection (thus the level of motor responses coincides with the stage in which response conflict emerges). In this kind of tasks, the possible responses are only two or four (the same 2 or 4 responses are repeated in the different trials) and they are typically button presses, thus critical variables are not likely to affect other (late) motor-related stages, that is, motor execution (such as happens instead with unpredictable, complex movements). Response selection coincides with the activation /inhibition of the motor codes of these responses.”***

RESPONSE: Because of Reviewer’s comment, we removed the sentence from the revised manuscript (see p. 55).
